# Investigating a pathogenic role for TXNDC5 in rheumatoid arthritis

**DOI:** 10.1186/ar3429

**Published:** 2011-07-29

**Authors:** Xiaotian Chang, Yan Zhao, Xinfeng Yan, Jihong Pan, Kehua Fang, Lin Wang

**Affiliations:** 1 National Laboratory for Bio-Drugs of Ministry of Health, Provincial Laboratory for Modern Medicine and Technology of Shandong, Research Center for Medicinal Biotechnology, Shandong Academy of Medical Sciences, Jingshi Road 18877, Jinan, Shandong, 250062. P. R. China; 2 Orthopedic Surgery Center of Shandong Qianfoshan Hospital. Jingshi Road 16766, Jinan, Shandong, 250014. P. R. China

## Abstract

**Introduction:**

Expression of TXNDC5, which is induced by hypoxia, stimulates cell proliferation and angiogenesis. Our previous study detected increased TXNDC5 expression in the synovial tissues of rheumatoid arthritis (RA) patients using proteomic methods. The current study investigated a pathogenic role for TXNDC5 in RA.

**Method:**

Expression of TXNDC5 in synovial membranes was quantitatively analyzed by immunohistochemistry, Western blotting and real-time polymerase chain reaction (PCR). Serum TXNDC5 levels and serum anti-TXNDC5 antibody levels were determined using sandwich enzyme-linked immunosorbent assay (ELISA). A total of 96 single nucleotide polymorphisms (SNPs) in or near the TXNDC5 gene were genotyped using custom-designed Illumina 96-SNP VeraCode microassay. Allele frequencies and genotype frequencies of SNPs were assessed using a case-control design in a cohort of 267 Chinese patients with RA, 51 patients with ankylosing spondylitis (AS) and 160 healthy controls. Additional genotyping of 951 patients with RA and 898 healthy controls was performed for four SNPs (rs2277105, rs369086, rs443861 and rs11962800) using the TaqMan method.

**Results:**

Real-time PCR, Western blotting and immunohistochemistry detected significantly higher TXNDC5 expression in the synovial tissues of RA patients compared to samples from patients with osteoarthritis (OA) or AS. ELISA detected significantly higher levels of TXNDC5 in the blood of RA patients compared to OA, AS and systemic lupus erythematosus patients, and healthy controls. ELISA did not detect significantly different levels of anti-TXNDC5 antibody in the blood of RA, OA and AS patients and healthy controls. A total of 9 SNPs (rs9505298, rs41302895, rs1225936, rs1225938, rs372578, rs443861, rs408014, rs9392189 and rs2743992) showed significant association with RA, while 16 SNPs (rs1044104, rs1225937, rs1225938, rs372578, rs89715, rs378963, rs1225944, rs1225947, rs1238994, rs369086, rs408014, rs368074, rs1225954, rs1225955, rs13209404 and rs3812162) showed significant association with AS. Taqman SNP assay demonstrated that rs443861 has an association with RA, which correlates with the microassay results.

**Conclusions:**

TXNDC5 is up-regulated in synovial tissues of RA patients. TXNDC5 has a genetic effect on the risk of RA and AS.

## Introduction

The thioredoxin domain, containing five (TXNDC5) proteins, also named ERp46, has a protein disulfide isomerase (PDI) domain that exhibits a high sequence similarity to thioredoxin, a catalyst of the rate limiting reaction of disulphide bond formation, isomerisation and reduction [1,2]. Yeast complementation tests showed that TXNDC5 can conduct PDI functions *in vivo *[3]. Indirect immunofluorescence microscopy and subcellular fractionation studies have shown that TXNDC5 is present both in the endoplasmic reticulum and the plasma membrane [4]. TXNDC5 is highly expressed in endothelial cells during hypoxic conditions, and plays important roles in anti-oxidative injury, anti-anoxia-induced apoptosis and the promotion of cell proliferation [1,2].

Abnormal proliferation of synovial fibroblasts and increased angiogenesis are pathological characteristics of rheumatoid arthritis (RA), an autoimmune disease that results in inflammation of the joints [5]. Using a proteomics approach, we detected increased TXNDC5 expression in synovial tissues from RA patients [6]. Furthermore, we detected significantly elevated levels of TXNDC5 in the synovial fluid of patients with RA [6]. RA is thought to decrease the oxygen supply, leading to synovial hypoxia and hypoperfusion [7,8]. Hence, we believe that up-regulation of TXNDC5 may play an important role in the pathogenesis of RA in the hypoxic environment.

In the current study, we quantitatively analyzed the expression of TXNDC5 in synovial tissues on both transcriptional and translational levels. We also examined TXNDC5 levels in the blood of RA patients using sandwich ELISA. To determine genetic effects of TXNDC5 on RA, we conducted Illumina GoldenGate assays to identify potential associations between TXNDC5 polymorphisms and RA. SNPs, including tag SNPs, SNPs in promoter regions, SNPs in untranslational regions (UTRs), SNPs in exons and SNPs within proximity to exons of the TXNDC5 gene were genotyped in RA populations, and potential associations were determined by case-control study and haplotype analysis.

## Materials and methods

### Sample collection of synovial tissues and blood

Synovial tissue samples were collected during knee joint replacement surgery from patients with RA (*n *= 10, 25 female, 23 to 70 years old, mean 50) and patients with osteoarthritis (OA) (*n *= 10, 6 female, 41 to 77 years old, mean 60). Synovial tissue samples from patients with AS (*n *= 10, 3 female, 28 to 54 years old, mean 35) were collected during hip joint replacement surgery. The diagnosis of RA was made according to the criteria of the American College of Rheumatology. The patients with RA had disease durations of 3-to-10 years and were classified as having erosive RA (Larsen class IV to V). They had high levels of C-reactive protein (30 to 100 mg/L, mean 24 mg/L), anti-CCP (300 to 3,000 U/ml) and RF (160 to 2,560 U/ml). AS patients had an average disease duration of seven years and were positive for HLA-B27 antigen. Their symptoms were consistent with the modified New York criteria for AS. Patients with AS and RA took disease-modifying antirheumatic drugs (DMARDs) before surgery. Patients with AS, RA and OA were also medicated with non-steroidal anti-inflammatory drugs (NSAIDs), which help reduce the pain and swelling of the joints, and decrease stiffness. All of AS and RA patients got treatment with DMARDs. Thus, the medical pretreatment does not influence the results and the experimental results are comparable. Additional file 1 in the supplementary materials summarizes the epidemiological data. All AS, RA and OA patients got treatment with NSAIDs. Synovial samples were dissected from connective tissues and immediately stored at -80°C until used.

Peripheral blood samples were collected from patients with RA (*n *= 267, 183 female) and AS (*n *= 51, 10 female). RA patients had a mean age of 51.7 years, while AS patients had a mean age of 35.9 years. The diagnosis of RA and AS was conducted as described above. Patients were selected from the same population living in the Shandong area of Northern China. A total of 160 (58 female) healthy individuals with a mean age of 48.0 years were blood donors; they did not have any personal or family history of serious illness. Control individuals were frequency matched to the expected age distribution of the cases and were from the same geographical area. Blood samples were put into Monovette tubes containing 3.8% sodium citrate.

Both patients and healthy controls gave their written consent to participate in the study and to allow their biological samples to be genetically analyzed. The Ethical Committee of Shandong Academy of Medicinal Sciences approved this study.

### Western blot analysis

Tissue samples weighing 200 μg from RA, OA and AS patients were homogenized in Cell Lysis Solution (Sigma-Aldrich, St. Louis, MO, USA) and centrifuged at 16,000 × g for five minutes at 4°C. Supernatants were collected after centrifugation, and protein concentrations were determined using the BCA Protein Assay Kit (Thermo Fisher Scientific, Rockford, IL, USA). Total protein was separated by sodium dodecyl sulphate polyacrylamide gel electrophoresis (SDS-PAGE) and trans-blotted onto nitrocellulose membranes (GE Healthcare, Piscataway, NJ, USA). Western blot analysis was conducted using anti-TXNDC5 antibody (Abcam, Cambridge, Cambridgeshire, UK)) at a 2,000-fold dilution. The antibody was raised in goats using an oligopeptide (SLHRFVLSQAKDEL) against TXNDC5. All primary and secondary antibodies were diluted in 5% nonfat dry skim milk in TBST (Tris base 0.02 M, NaCl 0.137 M in distilled water (pH 7.6), containing 0.1% Tween-20). Immunoreactive signals were detected with alkaline phosphatase-conjugated secondary antibodies and visualized using a Western blotting luminol reagent (GE Healthcare). Western blot images were acquired on a Typhoon Trio (GE Healthcare). Quantification was conducted using ImageQuant 5.2 software. Another membrane prepared by the same protocol was probed with anti-GADPH antibody (Santa Cruz Biotechnology, Santa Cruz, CA, USA) to normalize sample loading.

### Immunohistochemistry

Tissue sections of synovial tissues from RA, OA and AS patients were de-paraffinized and re-hydrated by standard procedures. Before the anti-TXNDC5 antibodies were applied, tissue sections were heated at 95°C for 10 minutes in citrate buffer solution (Sigma) for antigen recovery and then incubated with an endogenous peroxidase inhibitor (Maixin-Bio, Fuzhou, Fujian, China) for 30 minutes at room temperature. After washing with PBS buffer (NaCl 0.132 M, K2HPO4 0.0066 M, KH2PO4 0.001 5 M in distilled water, pH 7.6), sections were incubated with antibodies directed against TXNDC5 (Abcam) overnight at 4°C. Immunoreactions were processed using the UltraSensitive TM S-P Kit (Maixin-Bio) according to the manufacturer's instructions. Immunoreactive signals were visualized using DAB substrate, which stains the target protein yellow. Cell structures were counterstained with hematoxylin.

In order to determine antibody specificity and optimize antibody dilution, the tissue samples were incubated (1) with goat pre-immune serum (Maixin-Bio, China) or (2) treated by the modification buffer without addition of antibody.

### Immunofluorescent labeling

Tissue sections were processed as described above. After three washes with PBS buffer, tissue sections were treated with goat pre-immune serum (Maixin-Bio, China) for 30 minutes to improve the specificity of the immunoreaction. Slides were incubated with anti-TXNDC5 antibody (Abcam) at 4°C for 12 h and then washed with PBS. TRITC 5-conjugated anti-goat IgG (Sigma-aldrich) was added to the slides, and slides were incubated for 40 minutes at room temperature. Immunofluorescence was conducted with a Nikon 50i fluorescence microscope (Nikon, Shinjuku, Tokyo, Japan). To determine antibody specificities and optimize antibody dilutions, a series of control slides were prepared as follows: primary antibodies only, secondary antibodies only and normal goat serum only. Expression levels of TXNDC5 were evaluated with SimplePCI (Hamamatsu Photonics, Sewickley, PA, USA), a semi-quantitative scoring system that analyzes the results of immunofluorescent labeling according to signal density.

### Real-time PCR

Total RNA was isolated from the synovial tissues of RA, OA and AS patients using Trizol solution (Invitrogen Life Technologies, Carlsbad, California, USA) according to the manufacturer's protocol. Extracted total RNA was reverse-transcribed in a final volume of 10 μl using a RNA PCR Kit (TaKaRa, Katsushika, Tokyo, Japan). Real-time PCR reactions were conducted using the LightCycler 480 Instrument (Roche Molecular Biochemicals, Basel, Switzerland) and performed according to the manufacturer's protocol. Reactions were performed in a total volume of 10 ul, containing 1 ul of cDNA, 5 ul of SYBR Green Real-time PCR Master Mix (ToYoBo, Tokyo, Japan) and 1 ul of each primer. PCR amplification cycles were carried out as follows: 10 s at 95°C, 40 cycles of 5 s at 95°C and 31 s at 60°C. For each sample, two reactions were performed at the same time. One reaction was performed to determine the mRNA level of the target gene, and the second was performed to determine level of β-actin. The experiment was performed in triplicate. PCR products were confirmed by melt curve analysis. Relative mRNA expression was calculated using the comparative threshold cycle (Ct) method according to the following formula: Ratio = 2-ΔΔCt = 2-ΔCt(sample), where ΔCt = Ct of target genes - Ct of endogenous control gene (β-actin). The relative target gene expression was normalized in comparison to β-actin mRNA levels. Primer sequences for the amplification of human TXNDC5 were as follows: forward primer for TXNDC5, 5'-GGGTCAAGATCGCCGAAGTA-3'; reverse primer for TXNDC5, 5'-GCCTCCACTGTGCTCACTGA-3'; forward primer for human β-actin, 5'-TGGCACCCAGCACAATGAA-3'; and reverse primer for human β-actin, 5'-CTAAGTCATAGTCCGCCTAGAAGCA-3'. Primer efficiency was determined by serially diluting a standard RT reaction product. PCR efficiency was automatically calculated according to the dilution curve by the instrument software. Primer specificity was determined by both gel electrophoresis and melt curve analysis. Levels of TXNDC5 are expressed as the median and range. Statistical differences were assessed using the Mann-Whitney U-test; *P *< 0.05 was considered statistically significant.

### Sandwich ELISA detecting serum levels of TXNDC5

Blood was collected from patients with RA (*n *= 96, 75 females, 23 to 71 years old, mean 46), OA (*n *= 56, 16 females, 50 to 86 years old, mean 62), AS (*n *= 56, 19 females, 28 to 51 years old, mean 34) and systemic lupus erythematosus (SLE *n *= 56, 43 females, 23 to 73 years old, mean 40) as well as healthy controls (*n *= 48, 24 female, 20 to 40 years old, mean 31). Blood samples were collected using vacuum blood collection tubes. Following centrifugation at 1,000 × g for 30 minutes, serum was collected and stored at -80°C until use. We raised antibodies in rabbits using an oligopeptide (RDGKKVDQYKGKRD) conjugated to keyhole limpet hemocyanin (KLH). The specificity of the antibody was confirmed by Western blot analysis using various recombinant proteins. The antibody was compared with the antibody made by Abcam, which showed similar results of immunohistochemistry and Western blotting. Rabbit antibody was diluted 5,000-fold in 0.05 M carbonate-bicarbonate buffer (pH 9.6) and used to coat 96-well ELISA microplates (Corning Life Science, Amsterdam, Netherlands) by overnight incubation at 4°C. After a brief wash with PBS containing 0.1% Tween-20 (PBST), plates were blocked with 5% nonfat dry milk for one hour at room temperature. Next, blood samples were diluted 10-fold, and incubated in the plates for two hours at room temperature. After washing with PBST, goat anti-TXNDC5 antibody (Abcam), diluted 4,000-fold, was added to the plates and incubated for two hours at room temperature. Following a washing step, a 15,000-fold dilution of anti goat IgG alkaline phosphatase-conjugated antibody (Sigma) was added, and plates were incubated for 30 minutes at room temperature. Following another PBST wash, plates were developed by adding alkaline phosphatase yellow (pNPP) liquid substrate for ELISA (Sigma). Absorbance at 405 nm was measured using a plate reader (Synergy HT, Bio-Tek, Winooski, VT, USA). We repeated the ELISA three times and obtained the similar results.

Sandwich ELISA has low inter-assay and intra-assay variability and provides more accurate results than direct ELISA in which patient sera were coated on the plate and were then detected using the antibody.

### ELISA detecting serum levels of anti TXNDC5 antibody

Levels of anti TXNDC5 antibody were measured in the blood of patients with RA, OA, or AS (*n *= 50 for each disease) as well as healthy controls (*n *= 50). One hundred microLs of SLHRFVLSQAKDEL (0.5 ug/ul), the oligopeptide against TXNDC5, were coated onto 96-well ELISA microplates by overnight incubation at 4°C. After a brief wash with PBST, plates were blocked with 5% nonfat dry milk for one hour at room temperature. Serum samples, diluted 20-fold, were added and plates were incubated for two hours at 37°C. After washing with PBST, a 5,000-fold dilution of anti-human IgG alkaline phosphatase-conjugated antibody (Sigma) was added, and plates were incubated for 30 minutes at room temperature. Following another PBST wash, plates were developed by adding the alkaline phosphatase yellow (pNPP) liquid substrate for ELISA (Sigma). Absorbance at 405 nm was measured using a plate reader.

### Genomic DNA extraction

Genomic DNA was extracted from peripheral blood leukocytes using the DNA Blood Mini Kit from Qiagen (Hilden, Germany) according to the manufacturer's guidelines. Briefly, 5 ml of blood was mixed with triton lysis buffer (0.32 M sucrose, 1% Triton X-100, 5 mM MgCl_2_, H_2_O, 10 mM Tris-HCl, pH 7.5). Leukocytes were spun down and washed with H_2_O. Pellets were incubated with proteinase K at 56°C and subsequently salted out at 4°C using a substrate NaCl solution. Precipitated proteins were removed by centrifugation. The DNA in the supernatants was precipitated with ethanol, and the resulting DNA pellets were dissolved in 400 μl H_2_O.

### SNPs selection

Illumina GoldenGate assays were performed to genotype 96 SNPs within or near the TXNDC5 gene in 267 RA patients, 51 AS patients and 160 healthy control individuals from the Shandong area of North China. Tag SNPs, SNPs in untranslational region (UTR) and SNPs either in exons or in close proximity to exons of the gene encoding TXNDC5 were selected for genotyping. Tag SNPs were selected from HapMap data with a pair-wise r^2 ^≥0.8 and minor allele frequencies (MAF) over 0.05 [9,10]. Coding SNPs, SNPs near exons in 500 bp, SNPs in UTR and SNPs near the 5' and 3' ends of the gene were also selected. A total of 156 SNPs were candidates for Illumina's GoldenGate design and were submitted to Illumina for a design score. The Illumina Assay Design Tool (Illumina, San Diego, CA, USA) filtered out SNPs not suitable for the Illumina platform, such as insertions/deletions, tri- and tetra-allelic SNPs, and SNPs that are not uniquely localized. Finally, 96 SNPs with a design score of 1, spanning 0.18 Mb of the chromosome were selected. These SNPs included 5 coding SNPs, 4 SNPs at the 3' UTR, 35 tag SNPs and 53 SNPs in introns or near the 5' end. The gene information of these SNPs is shown in Table 1.

**Table 1:** Single nucleotide polymorphism (SNP) information

SNP ID	Chromosome position	Locus	Allele	Protein residue	
rs1044104	7881311	3' near gene	C/T		
rs9505298	7881449	3' near gene	A/G		
rs41302895	7881754	3' UTR	A/T		
rs1043784	7881931	3' UTR	A/G		
rs7764128	7882205	3' UTR	A/G		
rs8643	7883073	3' UTR	A/G		
rs9502656	7883386	synonymous	T	Asp [D]	
rs35264740	7883865	intron	C/T		
rs17764309	7883916	intron	A/G		
rs17696707	7884242	intron	A/G		
rs35871461	7884291	intron	C/T		
rs2277105	7884652	synonymous	A	Ala [A]	tag SNP
rs1225936	7885184	intron	A/C		
rs1225937	7885302	intron	C/T		
rs35794653	7885337	intron	-/A		
rs9505300	7885364	intron	C/T		
rs1225938	7886534	intron	A/G		
rs34342519	7886673	intron	-/C		
rs11962800	7886905	intron	A/G		
rs9505301	7887131	intron	A/G		
rs372578	7887223	intron	A/G		
rs7740689	7888066	intron	A/G		
rs89715	7888168	intron	C/T		
rs7745225	7888251	intron	C/T		
rs378963	7888328	intron	C/T		
rs45441296	7889033	missense	A	Met [M]	
rs1225944	7889088	intron	C/T		
rs34782746	7889254	intron	C/T		
rs1225946	7889465	intron	C/T		
rs7746818	7889466	intron	A/G		
rs34228534	7889773	frame shift		Gln [Q]	
rs1225947	7890121	intron	G/T		
rs13873	7891160	intron	G/T		tag SNP
rs34963444	7891384	intron	C/T		
rs7771314	7891403	intron	C/T		
rs9502657	7891682	intron	A/C		
rs9502658	7891947	synonymous	T	Phe [F]	
rs35365768	7892037	intron	-/C		
rs1225950	7892143	intron	C/G		
rs7749719	7894695	intron	C/T		
rs1238994	7894794	intron	G/T		
rs35650329	7895782	intron	-/G		
rs443861	7896491	intron	A/G		tag SNP
rs369086	7898875	intron	A/G		tag SNP
rs408014	7899394	intron	A/G		
rs368074	7899569	intron	C/G		
rs420970	7899651	intron	C/T		
rs1225954	7900028	intron	A/G		
rs1225955	7900709	intron	A/G		
rs6933089	7900856	intron	C/T		
rs13209404	7909967	intron	C/T		
rs13210097	7911345	5' near gene	A/C		
rs9502663	7911474	5' near gene	A/C		
rs3812162	7911702	5' near gene	A/C		tag SNP
rs34066135	7911855	5' near gene	-/G		
rs1632346	7913546	intron	C/T		tag SNP
rs1743634	7916207	intron	A/T		tag SNP
rs9505309	7917528	intron	G/T		tag SNP
rs6922018	7918311	intron	A/G		tag SNP
rs6923488	7918405	intron	C/T		tag SNP
rs1594467	7920361	intron	A/G		tag SNP
rs419588	7920808	intron	C/T		tag SNP
rs365936	7920904	intron	A/C		tag SNP
rs1237879	7932261	intron	A/G		tag SNP
rs627957	7936475	intron	C/T		tag SNP
rs155487	7938773	intron	A/G		tag SNP
rs10484327	7942566	intron	A/C		tag SNP
rs7764884	7970540	intron	A/G		tag SNP
rs7763447	7973380	intron	A/G		tag SNP
rs9406071	7974705	intron	C/T		tag SNP
rs6597292	7975259	intron	G/T		tag SNP
rs197119	7976745	intron	A/G		tag SNP
rs6597293	7987883	intron	C/G		tag SNP
rs11754300	7988766	intron	C/T		tag SNP
rs7744601	7988910	intron	C/T		tag SNP
rs2567226	7993977	intron	A/G		tag SNP
rs12204273	8002705	intron	A/G		tag SNP
rs9392182	8009035	intron	A/T		tag SNP
rs2207720	8019197	intron	C/T		tag SNP
rs9392189	8021532	intron	A/G		tag SNP
rs2815128	8023462	intron	G/T		tag SNP
rs2815142	8043546	intron	A/G		tag SNP
rs2743992	8054722	intron	A/G		tag SNP
rs2294436	8057688	intron	C/T		tag SNP
rs2743991	8060175	intron	A/G		tag SNP
rs9405369	8062437	intron	A/T		
rs12207627	8062532	intron	A/G		
rs2743989	8064035	intron	C/T		
rs2815153	8064050	intron	C/T		
rs2815154	8064084	intron	C/T		
rs9328453	8065127	5' near gene	A/G		
rs2815155	8065230	5' near gene	C/T		
rs12660697	8065707	5' near gene	A/G		
rs9392956	8065769	5' near gene	C/T		
rs9392957	8065781	5' near gene	A/C		
rs9505351	8066286	5' near gene	G/T		

### Genotyping using microarray

We performed genotyping using custom-designed Illumina 96-SNP VeraCode microarrays (Illumina). Genotyping was completed by technique service in Dr. Zhang Feng's Laboratory of he Beijing Institute of Genomics. A BeadXpress Reader using Illumina VeraCode GoldenGate Assay Kit was used. A total of 500 ng of sample DNA was used per assay. Genotype clustering and calling were performed using BeadStudio software (Illumina).

### Genotyping using Taqman SNP assay

Four tag SNPs, rs2277105, rs369086, rs443861 and rs11962800, were genotyped using TaqMan SNP genotyping assays in a cohort of 950 patients with RA (693 female) and 900 healthy controls (630 female). RA patients had a mean age of 46.2 years and were from the Shandong area of Northern China. The diagnosis of RA was conducted as described above. Healthy individuals with a mean age of 43.1 years were selected from the same geographical area. Assays were run on a LightCyclerH 480 Instrument (Roche) and evaluated according to the manufacturer's instructions. Reactions were carried out in a total volume of 10 μl using the following amplification protocol: denaturation at 95°C for 10 minutes, followed by 40 cycles of denaturation at 92°C for 15 seconds and finishing with annealing and extension at 60°C for 1 minute. The genotype of each sample was determined by measuring allele-specific fluorescence using SDS 2.3 software for allelic discrimination (Roche). Duplicate samples and negative controls were included to check the accuracy of genotyping.

### Statistical analysis

Genotyping SNPs were analyzed for association by comparison of the MAF in cases and controls. Associations of SNPs with RA and AS were evaluated using odds ratios (OR) with 95% confidence intervals (CI). Fisher's exact test was used for comparison between categorical variables. *P*-values less than 0.05 were considered statistically significant. After genotyping, SNP markers were evaluated for significant deviation from Hardy-Weinberg equilibrium. Calculations were performed using SHEsis and Haploview, two powerful web-based platforms for analyses of linkage disequilibrium, haplotype construction and genetic association at polymorphism loci [11,12].

## Results

### TXNDC5 expression in the synovial membranes of RA patients

**Figure 1:**
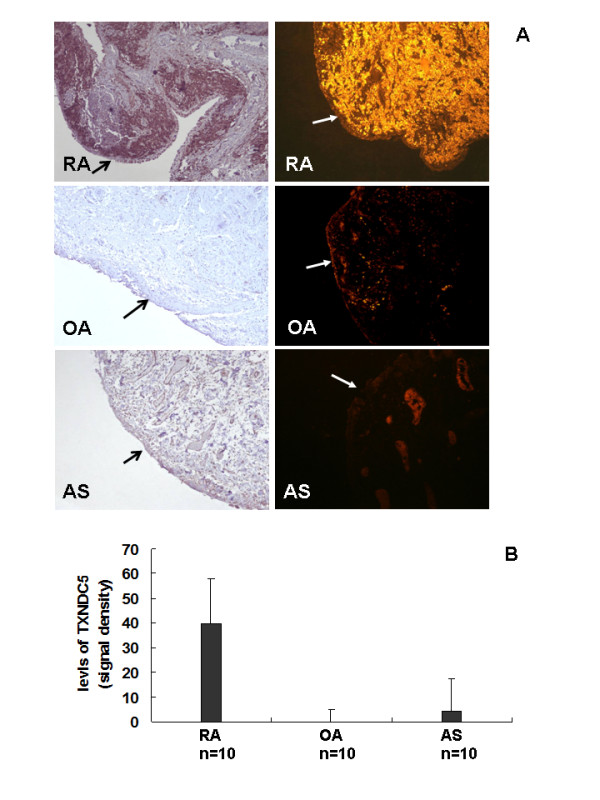
**Immunodetection of TXNDC5 in synovial membranes from patients with RA, OA and AS**. (**A**) Immunolocalization of TXNDC5 in synovial membranes. The left lane indicates results of immunohistochemistry, and the right lane indicates results of immunofluorescent labeling. Original magnification: 100×. Arrows indicate the upper layer of synovial membranes. (**B**) Semi-quantitative analysis of immunofluorescent signals of TXNDC5. TXNDC5 had significantly higher expression in the synovial tissue of RA patients compared to the synovial tissues of OA and AS patients. AS, ankylosing spondylitis; OA, osteoarthritis; RA, rheumatoid arthritis.

Immunohistochemistry analysis revealed significant TXCND5 expression in the thick lining layer and in many of the fibroblast-like cells of synovial membranes from RA patients (*n *= 10). Although detectable in the thin lining layer and some endothelial cells of small blood vessels, expression was very weak in the synovial membranes of OA patients (*n *= 10). In AS patients (*n *= 10), TXNDC5 expression was relatively low in synovial membranes and was mainly limited in endothelial cells of small blood vessels. These observations were confirmed by immunofluorescent labeling. Results are shown in Figure 1A. SimplePC (Hamamatsu Photonics, Sewickley, PA, USA), software designed to measure the signal density of the expression in a semi-quantitative manner, detected significantly higher levels of TXNDC5 in synovial tissues from RA patients compared to OA and AS patients (Figure 1B).

**Figure 2:**
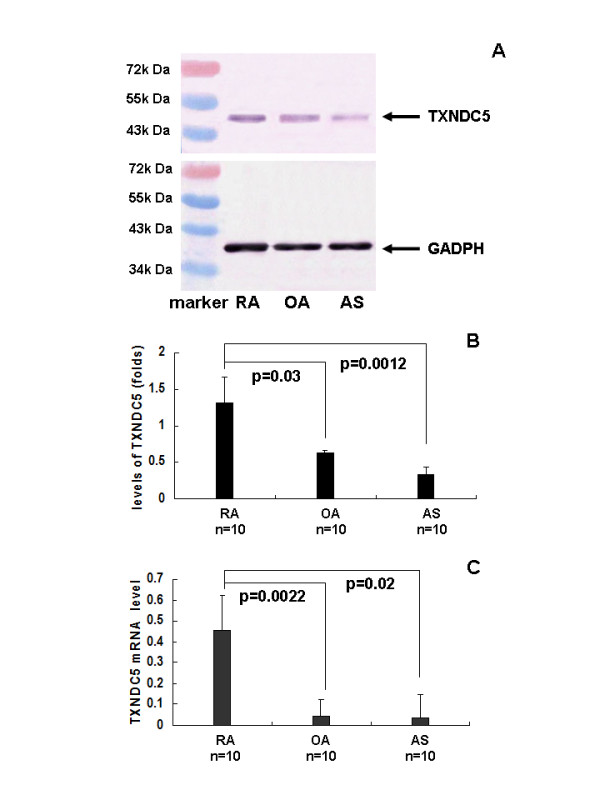
**Quantitative analysis of TXNDC5 expression**. (**A**) TXNDC5 at molecular weight of 50 kDa was detected in synovial tissues of RA, OA and AS patients using Western blot analysis. Sample loading was normalized using GADPH at molecular weight of 37 kDa. (**B**) TXNDC5 expression was semi-quantitatively analyzed by normalizing the signal density of TXNDC5 to that of GADPH. (**C**) TXNDC5 mRNA expression was measured in synovial tissues using real time PCR. The expression was normalized to that of β-actin. TXNDC5 had significantly higher expression in the synovial tissue of RA patients compared to the synovial tissues of OA and AS patients. AS, ankylosing spondylitis; OA, osteoarthritis; RA, rheumatoid arthritis.

Western blots revealed a protein with a molecular weight of 50 kDa. in each of the synovial tissues analyzed. Using GADPH as a reference, significantly increased TXNDC5 expression was detected in the synovial membranes of RA patients (*n *= 10), relative to the samples from OA (*n *= 10) and AS (*n *= 10) patients. These results were consistently observed in all of the synovial membranes examined (Figures 2A, B).

Transcription of TXNDC5 was quantified using real-time PCR. Similar to the Western blotting and immunolabeling results, all RA samples (*n *= 10) exhibited a higher degree of TXNDC5 mRNA expression compared to the OA (*n *= 10) and AS (*n *= 10) samples (Figure 2C). TXNDC5 was expressed at a low level in all OA samples.

### TXNDC5 levels in blood samples from RA patients

**Figure 3:**
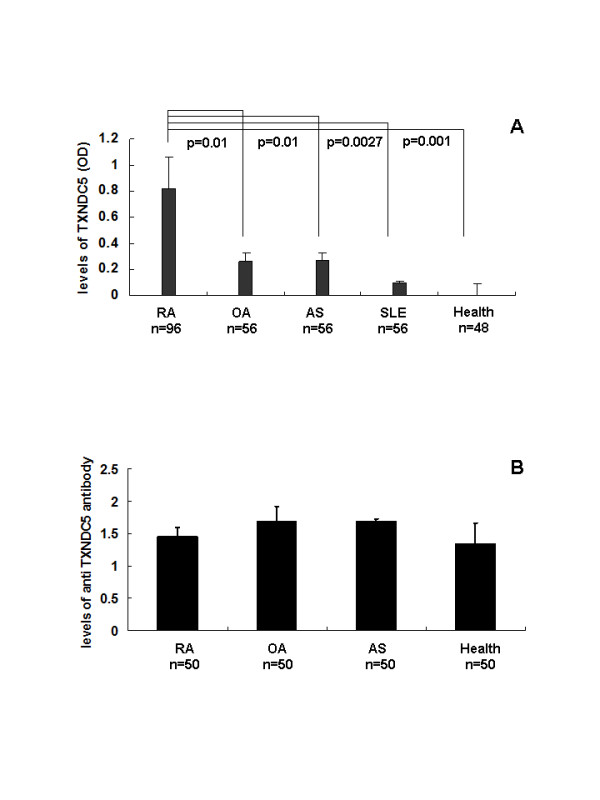
**Serum levels of TXNDC5 and anti-TXNDC5 antibody in patients with arthritic diseases and healthy controls**. TXNDC5 levels are represented by OD values of absorbance at 405 nm and are expressed as the mean ± standard error of the mean. (**A**) A sandwich ELISA detected increased level of TXNDC5 in blood samples from RA patients compared to samples from OA, AS and SLE patients, as well as from healthy controls. (**B**) An ELISA indicated that levels of anti-TXNDC5 antibodies were not significantly different among blood samples from RA, OA and AS patients and the healthy controls. AS, ankylosing spondylitis; OA, osteoarthritis; RA, rheumatoid arthritis.

A sandwich ELISA was used to measure levels of TXNDC5 in the blood of RA patients with chronic inflammation. Levels of TXNDC5 were significantly increased in samples from RA patients compared to samples from OA, AS and SLE patients. Serum TXNDC5 expression in RA patients was also significantly elevated compared to healthy controls (Figure 3A). An ELISA was used to measure serum anti-TXNDC5 antibody levels of the patients. These were not significantly different from serum levels from RA, OA, and AS patients and healthy controls (Figure 3B).

### Genotyping of SNPs located in TXNDC5

We genotyped 96 SNPs across the TXNDC5 gene from 267 Han Chinese patients with RA, 51 patients and 160 control individuals. All SNPs yielded genotype data, and the study sample success rate was 99.1%. Differences in allele frequencies and genotype frequencies between cases and controls were compared. Overall, nine SNPs (rs9505298, rs41302895, rs1225936, rs1225938, rs372578, rs443861, rs408014, rs9392189 and rs2743992) were found to be significantly associated with RA (*P *< 0.05). A total of 16 SNPs (rs1044104, rs1225937, rs1225938, rs372578, rs89715, rs378963, rs1225944, rs1225947, rs1238994, rs369086, rs408014, rs368074, rs1225954, rs1225955, rs13209404 and rs3812162) were found to be significantly associated with AS (*P *< 0.05). Among the genotyped SNPs, three SNPs (rs1225938, rs372578 and rs408014) had significant association with both RA and AS. All SNPs retained in the analysis were in Hardy-Weinberg equilibrium (*P *> 0.05) in the overall samples. The allele and genotype frequencies of the associated SNPs between cases and controls are shown in Tables 2 and 3. Other SNPs of the TXNDC5 gene polymorphisms did not disclose significant differences in allelic frequencies and genotype frequencies between the RA patients and controls or between AS patients and controls.

**Table 2:** Allele and genotype frequencies in a case-control cohort of patients with RA

dbSNP identity	Allele/ Genotype	Numbers of patients with RA (%)	Numbers of controls (%)	Fisher's *P-*value	Odds ratio (95% CI)
rs9505298	A	75 (0.144)	10 (0.032)	1.83E-07	5.157303 (2.624041 to 10.136190)
	G	445 (0.856)	306 (0.968)		5.157303 (2.624041 to 10.136190)
	AA	1 (0.004)	0 (0.000)	3.13E-07	
	AG	73 (0.281)	10 (0.063)		
	GG	186 (0.715)	148 (0.937)		
rs41302895	A	64 (0.120)	9 (0.028)	3.32E-06	4.725546 (2.317854 to 9.634252)
	T	468 (0.880)	311 (0.972)		4.725546 (2.317854 to 9.634252)
	AA	1 (0.004)	0 (0.000)	9.11E-06	
	AT	62 (0.233)	9 (0.056)		
	TT	203 (0.763)	151 (0.944)		
rs1225936	A	24 (0.045)	2 (0.006)	0.001438	7.494071 (1.759029 to 31.927328)
	C	506 (0.955)	316 (0.994)		7.494071 (1.759029 to 31.927328)
	AC	24 (0.091)	2 (0.013)	0.001201	7.817427 (1.821928 to 33.542572)
	CC	241 (0.909)	157 (0.987)		7.817427 (1.821928 to 33.542572)
rs1225938	A	270 (0.509)	190 (0.594)	0.016879	0.710526 (0.536647 to 0.940745)
	G	260 (0.491)	130 (0.406)		0.710526 (0.536647 to 0.940745)
	AA	53 (0.200)	52 (0.325)	0.013717	
	AG	164 (0.619)	86 (0.537)		
	GG	48 (0.181)	22 (0.138)		
rs372578	A	224 (0.424)	109 (0.341)	0.015688	1.426364 (1.068889 to 1.903392)
	G	304 (0.576)	211 (0.659)		1.426364 (1.068889 to 1.903392)
	AA	45 (0.170)	21 (0.131)	0.029497	
	AG	134 (0.508)	67 (0.419)		
	GG	85 (0.322)	72 (0.450)		
rs443861*	A	117 (0.221)	48 (0.150)	0.011538	1.605327 (1.109766 to 2.322179)
	G	413 (0.779)	272 (0.850)		1.605327 (1.109766 to 2.322179)
	AA	6 (0.023)	3 (0.019)	0.016509	
	AG	105 (0.396)	42 (0.263)		
	GG	154 (0.581)	115 (0.719)		
rs408014	A	303 (0.574)	211 (0.659)	0.013531	0.695671 (0.521353 to 0.928274)
	G	225 (0.426)	109 (0.341)		0.695671 (0.521353 to 0.928274)
	AA	86 (0.326)	72 (0.450)	0.03402	
	AG	131 (0.496)	67 (0.419)		
	GG	47 (0.178)	21 (0.131)		
rs9392189*	A	116 (0.221)	103 (0.322)	0.001239	0.598991 (0.438317 to 0.818563)
	G	408 (0.779)	217 (0.678)		0.598991 (0.438317 to 0.818563)
	AA	16 (0.061)	18 (0.113)	0.007146	
	AG	84 (0.321)	67 (0.419)		
	GG	162 (0.618)	75 (0.469)		
rs2743992*	A	230 (0.437)	163 (0.509)	0.041455	0.748425 (0.566338 to 0.989055)
	G	296 (0.563)	157 (0.491)		0.748425 (0.566338 to 0.989055)
	AA	43 (0.163)	44 (0.275)	0.022536	
	AG	144 (0.548)	75 (0.469)		
	GG	76 (0.289)	41 (0.256)		

160 controls and 266 cases were observed; *represents tag SNP

95% CI, 95% confidence interval; RA, rheumatoid arthritis; SNP, single nucleotide polymorphism

**Table 3:** Allele and genotype frequencies in a case-control cohort of patients with AS

dbSNP identity	Allele/Genotype	Numbers of patients with RA (%)	Numbers of controls (%)	Fisher's *P-*value	Odds Ratio (95% CI)
rs1044104	A	49 (0.480)	103 (0.322)	0.0037	1.947793 (.237327 to 3.066204)
	G	53 (0.520)	217 (0.678)		1.947793 (.237327 to 3.066204)
	AA	13 (0.255)	20 (0.125)	0.022555	
	AG	23 (0.451)	63 (0.394)		
	GG	15 (0.294)	77 (0.481)		
rs1225937	A	67 (0.657)	252 (0.787)	0.007503	0.516553 (0.316863 to 0.842090)
	G	35 (0.343)	68 (0.212)		0.516553 (0.316863 to 0.842090)
	AA	22 (0.431)	99 (0.619)	0.027945	
	AG	23 (0.451)	54 (0.338)		
	GG	6 (0.118)	7 (0.044)		
rs1225938	A	46 (0.451)	190 (0.594)	0.011468	0.562030 (0.358612 to 0.880834)
	G	56 (0.549)	130 (0.406)		0.562030 (0.358612 to 0.880834)
	AA	11 (0.216)	52 (0.325)	0.013973	
	AG	24 (0.471)	86 (0.537)		
	GG	16 (0.314)	22 (0.138)		
rs372578	A	50 (0.490)	109 (0.341)	0.006659	1.861327 (1.184652 to 2.924518)
	G	52 (0.510)	211 (0.659)		1.861327 (1.184652 to 2.924518)
	AA	14 (0.275)	21 (0.131)	0.029107	
	AG	22 (0.431)	67 (0.419)		
	GG	15 (0.294)	72 (0.450)		
rs89715	A	52 (0.510)	212 (0.662)	0.005544	0.529811 (0.337112 to 0.832662)
	G	50 (0.490)	108 (0.338)		0.529811 (0.337112 to 0.832662)
	AA	15 (0.294)	73 (0.456)	0.026647	
	AG	22 (0.431)	66 (0.412)		
	GG	14 (0.275)	21 (0.131)		
rs378963	A	63 (0.643)	253 (0.791)	0.002897	0.476680 (0.291090 to 0.780596)
	G	35 (0.357)	67 (0.209)		0.476680 (0.291090 to 0.780596)
	AA	20 (0.408)	100 (0.625)	0.012477	
	AG	23 (0.469)	53 (0.331)		
	GG	6 (0.122)	7 (0.044)		
rs1225944	A	35 (0.357)	66 (0.206)	0.002276	2.138047 (1.304553 to 3.504071)
	G	63 (0.643)	254 (0.794)		2.138047 (1.304553 to 3.504071)
	AA	6 (0.122)	7 (0.044)	0.010406	
	AG	23 (0.469)	52 (0.325)		
	GG	20 (0.408)	101 (0.631)		
rs1225947	A	51 (0.500)	108 (0.338)	0.0032	1.962963 (1.249087 to 3.084832)
	C	51 (0.500)	212 (0.662)		1.962963 (1.249087 to 3.084832)
	AA	13 (0.255)	21 (0.131)	0.017854	
	AC	25 (0.490)	66 (0.412)		
	CC	13 (0.255)	73 (0.456)		
rs1238994	A	52 (0.510)	210 (0.656)	0.007966	0.544762 (0.346807 to 0.855709)
	C	50 (0.490)	110 (0.344)		0.544762 (0.346807 to 0.855709)
	AA	15 (0.294)	71 (0.444)	0.031615	
	AC	22 (0.431)	68 (0.425)		
	CC	14 (0.275)	21 (0.131)		
rs369086*	A	35 (0.343)	66 (0.208)	0.005322	1.994573 (1.221370 to 3.257261)
	G	67 (0.657)	252 (0.792)		1.994573 (1.221370 to 3.257261)
	AA	6 (0.118)	7 (0.044)	0.022037	
	AG	23 (0.451)	52 (0.327)		
	GG	22 (0.431)	100 (0.629)		
rs408014	A	50 (0.500)	211 (0.659)	0.004146	0.516588 (0.327716 to 0.814312)
	G	50 (0.500)	109 (0.341)		0.516588 (0.327716 to 0.814312)
	AA	14 (0.280)	72 (0.450)	0.020441	
	AG	22 (0.440)	67 (0.419)		
	GG	14 (0.280)	21 (0.131)		
rs368074	C	50 (0.500)	109 (0.341)	0.004146	1.935780 (1.228031 to 3.051425)
	G	50 (0.500)	211 (0.659)		1.935780 (1.228031 to 3.051425)
	CC	14 (0.280)	21 (0.131)	0.020441	
	CG	22 (0.440)	67 (0.419)		
	GG	14 (0.280)	72 (0.450)		
rs1225954	A	50 (0.500)	109 (0.341)	0.004146	1.935780 (1.228031 to 3.051425)
	G	50 (0.500)	11 (0.659)		1.935780 (1.228031 to 3.051425)
	AA	14 (0.280)	21 (0.131)	0.020441	
	AG	22 (0.440)	67 (0.419)		
	GG	14 (0.280)	72 (0.450)		
rs1225955	A	50 (0.500)	108 (0.338)	0.003427	1.962963 (1.244946 to 3.095092)
	G	50 (0.500)	212 (0.662)		1.962963 (1.244946 to 3.095092)
	AA	14 (0.280)	21 (0.131)	0.018534	
	AG	22 (0.440)	66 (0.412)		
	GG	14 (0.280)	73 (0.456)		
rs13209404	A	30 (0.300)	59 (0.184)	0.013556	1.895884 (1.135508 to 3.165434)
	G	70 (0.700)	261 (0.816)		1.895884 (1.135508 to 3.165434)
	AA	4 (0.080)	7 (0.044)	0.043599	
	AG	22 (0.440)	45 (0.281)		
	GG	24 (0.480)	108 (0.675)		
rs3812162*	A	70 (0.700)	268 (0.838)	0.002475	0.452736 (0.268969 to 0.762060)
	C	30 (0.300)	52 (0.163)		0.452736 (0.268969 to 0.762060)
	AA	23 (0.460)	114 (0.713)	0.004576	
	AC	24 (0.480)	40 (0.250)		
	CC	3 (0.060)	6 (0.037)		

160 controls & 266 cases were observed; *represents tag SNP. 95% CI, 95% confidence interval; AS, ankylosing spondylitis; SNP, single nucleotide polymorphism.

**Figure 4:**
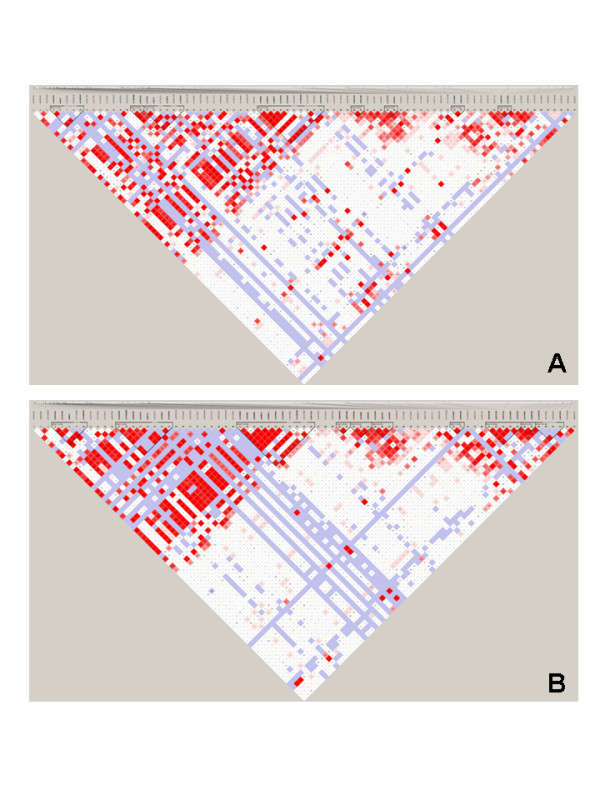
**LD plot of the 96 genotyped SNPs in the TXNDC5 gene**. (**A**) Linkage disequilibrium in the RA group. (**B**) Linkage disequilibrium in the AS group. Red areas representing higher levels of LD. Blue areas represent LD comparisons with low confidence of estimation. Dark triangles represent haplotype blocks. Numbers in squares are D' values. AS, ankylosing spondylitis; RA, rheumatoid arthritis; SNP, single nucleotide polymorphism.

Linkage disequilibrium (LD) analysis was performed within the tested SNPs. Pairwise D' values between all SNPs were calculated to determine the extent of LD. LD analysis defined eight blocks in TXNDC5 within the RA population. Rs372578, rs408014 and rs2743992, which showed strong association with RA, were in Blocks 3, 4 and 8, respectively. LD analysis defined 10 blocks in TXNDC5 within the studied AS population. Block 2 contained rs372578, rs89715, ra378963 and rs1225944, while Block 3 contained rs1238994, rs369086, rs408014 rs368074, rs1225954, rs1225955, rs13209404 and rs3812162, SNPs that showed strong association within AS patients. These results are shown in Figure 4A, B.

In the RA population, haplotype analysis defined 27 haplotypes (frequency > 1%) in the TXNDC5 gene by LD. Haplotype AA (frequency 79.3%) in Block 2, haplotype GAAG (frequency 56.4%) in Block 3, haplotypes GAGGGGA and AGCAAAC (frequencies 56.6% and 23.1%, respectively) in Block 4 and haplotype AG (frequency 79.3%) in Block 8 provide significant evidence to be associated with RA risk (*P *= 0.0446, 0.0125, 0.0112, 0.0081 and 0.0336, respectively). Haplotype analysis defined 40 haplotypes (frequency > 1%) within the RA cohort by LD in the control population. Haplotypes AAAGAAG and GAAAGGA (frequencies 44.1% and 33.3%, respectively) in Block 2, haplotypes AGGAGGGGA and CGAGCAAAC (frequencies 48.9% and 29.3%, respectively) in Block 3, haplotype GG (frequency 56.9) in Block 4, and haplotype AGA (frequency 4%) in Block 5 provided significant evidence to be associated with AS risk (*P *= 0.0198, 0.0043, 0.0044, 0.0018, 0.0187 and 0.0053, respectively). The haplotype frequencies in a case-control cohort of patients with RA and AS are shown in Tables 4 and 5. The raw microarray data were available as an Additional file 2 to perform association, LD and haplotype analysis.

**Table 4:** Haplotype frequencies in a case-control cohort of patients with rheumatoid arthritis

Haplotype	**Freq**.	Case, Control Ratio Counts	Case, Control Frequencies	*P-*Value
**Block 1**				
AGG	0.827	426.9: 101.1, 273.0: 45.0	0.808, 0.858	0.0625
GAA	0.145	81.0: 447.0, 42.0: 276.0	0.153, 0.132	0.3939
AAG	0.012	7.8: 520.2, 2.0: 316.0	0.015, 0.006	0.2649
**Block 2**				
AA	0.814	418.9: 109.1, 269.9: 48.1	0.793, 0.849	0.0446
GG	0.155	87.9: 440.1, 42.9: 275.1	0.166, 0.135	0.2187
AG	0.024	15.1: 512.9, 5.1: 312.9	0.029, 0.016	0.2441
**Block 3**				
GAAG	0.597	297.7: 230.3, 207.0: 111.0	0.564, 0.651	0.0125
AGGA	0.236	135.3: 392.7, 64.0: 254.0	0.256, 0.201	0.0674
AGAG	0.146	83.8: 444.2, 40.0: 278.0	0.159, 0.126	0.1902
AAAG	0.012	6.1: 521.9, 4.0: 314.0	0.012, 0.013	0.8831
**Block 4**				
GAGGGGA	0.6	296.8: 227.2, 207.0: 109.0	0.566, 0.655	0.0112
AGCAAAC	0.202	120.9: 403.1, 49.0: 267.0	0.231, 0.155	0.0081
GGCAAGA	0.149	83.0: 441.0, 42.0: 274.0	0.158, 0.133	0.3157
AGCAAGA	0.022	10.1: 513.9, 8.0: 308.0	0.019, 0.025	0.5599
AGCAAAA	0.017	6.0: 518.0, 8.0: 308.0	0.012, 0.025	0.1299
**Block 5**				
GG	0.669	344.6: 183.4, 220.9: 97.1	0.653, 0.695	0.2075
AA	0.213	112.6: 415.4, 67.6: 250.4	0.213, 0.213	0.9829
AG	0.117	69.7: 458.3, 29.4: 288.6	0.132, 0.092	0.0828
**Block 6**				
AA	0.715	380.5: 145.5, 222.8: 95.2	0.723, 0.701	0.4774
AG	0.18	88.7: 437.3, 63.5: 254.5	0.169, 0.200	0.2564
GG	0.1	53.3: 472.7, 31.5: 286.5	0.101, 0.099	0.9173
**Block 7**				
AA	0.699	374.8: 153.2, 216.9: 101.1	0.710, 0.682	0.3934
AG	0.214	110.9: 417.1, 70.1: 247.9	0.210, 0.220	0.7211
GG	0.085	41.1: 486.9, 30.9: 287.1	0.078, 0.097	0.3288
**Block 8**				
AG	0.531	294.0: 232.0, 153.8: 164.2	0.559, 0.484	0.0336
AA	0.376	187.8: 338.2, 129.2: 188.8	0.357, 0.406	0.1518
GA	0.092	42.9: 483.1, 34.8: 283.2	0.082, 0.109	0.1742

**Table 5:** Haplotype frequencies in a case-control cohort of patients with ankylosing spondylitis

Haplotype	**Freq**.	Case, control ratio counts	Case, control frequencies	*P-v*alue
**Block 1**				
AGGG	0.755	75.0: 27.0, 242.0: 76.0	0.735, 0.761	0.5994
GAAA	0.136	15.0: 87.0, 42.0: 276.0	0.147, 0.132	0.7006
AAGG	0.1	11.0: 91.0, 31.0: 287.0	0.108, 0.097	0.7616
**Block 2**				
AAAGAAG	0.541	45.0: 57.0, 182.2: 135.8	0.441, 0.573	0.0198
GAAAGGA	0.23	34.0: 68.0, 62.5: 255.5	0.333, 0.196	0.0043
GGGAGAG	0.126	13.0: 89.0, 40.0: 278.0	0.127, 0.126	0.9686
GAAGAAG	0.065	6.0: 96.0, 21.3: 296.7	0.059, 0.067	0.7731
**Block 3**				
AGGAGGGGA	0.609	49.7: 51.9, 206.0: 112.0	0.489, 0.648	0.0044
CGAGCAAAC	0.187	29.8: 71.8, 49.0: 269.0	0.293, 0.154	0.0018
CAGGCAAGA	0.134	14.3: 87.3, 42.0: 276.0	0.140, 0.132	0.829
CGAGCAAAA	0.022	1.2: 100.4, 8.0: 310.0	0.012, 0.025	0.4324
CGAGCAAGA	0.017	4.0: 97.6, 3.0: 315.0	0.039, 0.010	0.0408
CAAGCAAGA	0.012	0.0: 101.6, 5.0: 313.0	0.000, 0.016	0.2042
**Block 4**				
GG	0.664	58.0: 44.0, 221.0: 97.0	0.569, 0.695	0.0187
AA	0.228	28.0: 74.0, 67.7: 250.3	0.275, 0.213	0.1962
AG	0.108	16.0: 86.0, 29.3: 288.7	0.157, 0.092	0.067
**Block 5**				
AAA	0.523	45.9: 56.1, 173.7: 144.3	0.450, 0.546	0.0915
AGC	0.29	33.3: 68.7, 88.6: 229.4	0.327, 0.279	0.3548
GGC	0.097	7.6: 94.4, 33.0: 285.0	0.075, 0.104	0.3851
AAC	0.072	10.7: 91.3, 19.4: 298.6	0.105, 0.061	0.1339
AGA	0.013	4.1: 97.9, 1.3: 316.7	0.040, 0.004	0.0053
**Block 6**				
AAA	0.709	77.9: 24.1, 219.8: 98.2	0.764, 0.691	0.159
AGG	0.101	9.5: 92.5, 32.9: 285.1	0.093, 0.104	0.7639
GGG	0.096	9.4: 92.6, 30.9: 287.1	0.092, 0.097	0.8872
AGA	0.084	5.0: 97.0, 30.1: 287.9	0.049, 0.095	0.1475
**Block 7**				
AA	0.683	70.0: 32.0, 217.0: 101.0	0.686, 0.682	0.9415
AG	0.221	23.0: 79.0, 70.0: 248.0	0.225, 0.220	0.9096
GG	0.095	9.0: 93.0, 31.0: 287.0	0.088, 0.097	0.7819
**Block 8**				
GAAGA	0.49	53.9: 48.1, 151.8: 166.2	0.529, 0.477	0.3656
GAAAA	0.198	21.1: 80.9, 62.1: 255.9	0.206, 0.195	0.8077
AAAAG	0.14	14.0: 88.0, 44.9: 273.1	0.137, 0.141	0.9123
AAAAA	0.059	3.0: 99.0, 22.0: 296.0	0.029, 0.069	0.14
ACGAA	0.057	5.0: 97.0, 19.0: 299.0	0.049, 0.060	0.6831
AAGAA	0.042	4.0: 98.0, 13.9: 304.1	0.039, 0.044	0.8369
**Block 9**				
AT	0.517	53.0: 49.0, 164.0: 154.0	0.520, 0.516	0.9455
GA	0.28	26.6: 75.4, 91.0: 227.0	0.261, 0.286	0.6165
GT	0.203	22.4: 79.6, 63.0: 255.0	0.220, 0.198	0.6357
**Block 10**				
AG	0.802	78.2: 23.8, 258.5: 59.5	0.767, 0.813	0.3066
GA	0.161	18.7: 83.3, 48.8: 269.2	0.184, 0.153	0.4683
GG	0.032	3.8: 98.2, 9.5: 308.5	0.037, 0.030	0.7032

We performed additional genotyping for four SNPs (rs2277105, rs369086, rs443861 and rs11962800) in an independent case-control study using the TaqMan method. The study was conducted within 951 patients with RA and 898 healthy controls. Allelic frequencies and gene frequencies of the four tag SNPs did not deviate from Hardy-Weinberg equilibrium in both case and the controls. Allelic frequency of the tag SNPs was compared between RA patients and controls. Among the polymorphisms identified, the allele frequency and gene frequency for tag SNP rs443861 demonstrated statistically significant evidence for association with RA (*P *= 0.008320, and 0.010110. This SNP was also determined to have significant association with RA by Illumina 96-SNP VeraCode microarray. The tag SNPs of rs2277105, rs369086 and rs11962800 did not disclose significant differences in allelic frequencies and gene frequencies between RA patients and controls (Table 6).

**Table 6:** Allele and genotype frequencies in a case-control cohort of patients with RA

dbSNP identity	Allele/ Genotype	No. of patients with RA (%)	No. of controls (%)	Fisher's *P-*value
rs2277105	C	1581 (0.840)	1512 (0.848)	0.491098
	O(freq)	19 (0.010)	12 (0.007)	
	T	283 (0.150)	260 (0.146)	
	CC	670 (0.719)	650 (0.734)	0.549842
	CT	241 (0.259)	212 (0.239)	
	TT	21 (0.023)	24 (0.027)	
rs11962800	A	1568 (0.831)	1523 (0.853)	0.173781
	O(freq)	15 (0.008)	11 (0.006)	
	G	304 (0.161)	251 (0.141)	
	AA	656 (0.701)	656 (0.740)	0.184145
	AG	256 (0.274)	211 (0.238)	
	GG	24 (0.026)	20 (0.023)	
rs443861	A	357 (0.190)	275 (0.154)	0.00832
	O(freq)	19 (0.010)	12 (0.007)	
	G	1507 (0.800)	1497 (0.839)	
	AA	51 (0.055)	26 (0.029)	0.01011
	AG	255 (0.274)	223 (0.252)	
	GG	626 (0.672)	637 (0.719)	
rs369086	A	485 (0.257)	460 (0.258)	0.510123
	O(freq)	18 (0.010)	11 (0.006)	
	G	1381 (0.733)	1314 (0.736)	
	AA	61 (0.065)	71 (0.080)	0.260584
	AG	363 (0.389)	318 (0.359)	
	GG	509 (0.546)	498 (0.561)	

898 controls and 951 RA cases observed. RA, rheumatoid arthritis; SNP, single nucleotide polymorphism

## Discussion

In the present study, TXNDC5 expression was quantitatively assessed both at the transcriptional level and translational level. In comparison to synovial tissue samples from OA and AS patients, TXNDC5 expression was significantly increased in the synovial tissues of RA patients as determined by immunohistochemistry and Western blotting. Real time PCR also detected increased TXNDC5 mRNA levels in the synovial membranes of RA patients. Furthermore, sandwich ELISA detected increased expression of TXNDC5 in both the synovial fluid and blood of RA patients [6]. Taken together, these results confirm the increased expression of TXNDC5 in the synovium and blood of RA patients. In the present study, we did not detect increased levels of autoantibodies directed against TXNDC5 in the blood of RA patients, indicating that the over-expression of TXNDC5 does not directly cause an autoimmune response as an autoantigen like some citrullinated proteins [13]. We processed Western blotting with protein extracted from the whole synovial tissue. The immunohistochemistry focuses on the expression of TXNDC5 in the lining area and the deep lining area of the synovial membrane. Immunofluorescent immunocytochemistry semi-quantified the expression level in one tissue region rather than the whole tissue. In addition, synovial tissues of RA and AS have significantly increased angiogenesis in which endothelial cells of blood vessels have strong expression of TXNDC5. Thus, it is possible the result of semi-quantification of immunofluorescent immunocytochemistry is a little different from the result of Western blotting.

TXNDC5 expression is up-regulated by hypoxia and has a protective effect on endothelial cells by inducing folding and chaperone activity in hypoxia-induced anti-apoptotic molecules [1,2]. RA is thought to decrease the oxygen supply, leading to synovial hypoxia and hypoperfusion [7,8]. Using co-immunoprecipitation followed by mass spectrometry, Charlton *et al. *found that TXNDC5 interacts with the N-terminal residues of AdipoR1. Further, transient knockdown of TXNDC5 in HeLa cells increased the levels of AdipoR1 and AdipoR2, which correlated with the increased adiponectin-stimulated phosphorylation of AMPK. However, adiponectin-stimulated phosphorylation of p38MAPK was reduced following TXNDC5 knockdown [4]. Recent reports indicate that AdipoR1 and AdipoR2 mediate the insulin-sensitizing adipokine adiponectin. RA is associated with the increased production of adipokines, cytokine-like mediators that are produced mainly in adipose tissue and synovial cells [14]. Frommer *et al. *demonstrated that adiponectin was present in inflamed synovium at sites of cartilage invasion in lymphocyte infiltrates and in perivascular areas. Adiponectin stimulates synovial fibroblasts to secrete chemokines, proinflammatory cytokines, prostaglandin synthases, growth factors and factors for bone metabolism and matrix remodelling. This adiponectin-mediated effect was p38 MAPK and protein kinase C dependent. Adiponectin promotes inflammation through cytokine and chemokine production that attracts inflammatory and pro-destructive cells to the synovium, which, in turn promotes matrix destruction at sites of cartilage invasion [15]. Choi *et al. *reported that adiponectin might contribute to synovitis and joint destruction in RA by stimulating vascular endothelial growth factor, matrix metalloproteinase-1, and matrix metalloproteinase-13 expression in fibroblast-like synoviocytes [16]. Additionally, Tian *et al. *also reported that increased PDI activity in myocardial endothelial cells in mice stimulates angiogenesis under hypoxia condition [17]. These results support the possibility that the increase of TXNDC5 expression in the synovial tissues of RA patients stimulates the synovial ocular pannus, pro-inflammation and bone degradation. However, the detailed mechanism requires further investigation.

TXNDC5 is a newly identified member of this protein family. TXNDC5 has been genetically mapped to chromosome 6p24.3. The gene encoding TXNDC5 is approximately 845.2 k bp, and it is divided into 13 exons. The present study genotyped 96 SNPs flanking the TXNDC5 gene through Illumina GoldenGate assays. Further, the study also genotyped four tag SNPs in the TXNDC5 gene using the Taqman method to confirm association to RA in a large number of samples. Both methods revealed the strong association of rs443861 with RA, indicating a genetic effect of TXNDC5 on RA risk. Although the genetic data of the present study indicated the possible association of TXNDC5 to RA, not enough data support the idea that the increased expression was caused by a genetic mechanism. The increased expression of TXNDC5 could be induced by hypoxia in RA rather than genetic variation of the gene. To determine whether variations in the TXNDC5 gene contributed to the risk of developing nonsegmental vitiligo (NSV), Jeong *et al. *conducted a case-control association study within a Korean population. They genotyped seven SNPs and found that three exonic SNPs (rs1043784, rs7764128 and rs8643) were statistically associated with NSV. The haplotypes AGG and GAA, consisting of rs1043784, rs7764128 and rs8643, demonstrated a significant association with NSV [18]. Lin *et al. *reported that SNP rs13873 and haplotypes rs1225934 to rs13873 of BMP6-TXNDC5 genes were significantly associated with schizophrenia [19]. These reports indicate that TXNDC5 plays a role in the pathogenesis of other diseases. Our results demonstrated that rs1043784, rs7764128, rs1225934 and rs8643 were not significantly associated with RA and AS.

## Conclusions

Our study demonstrated significantly increased TXNDC5 expression in the synovium and blood of RA patients, which may contribute to the irregular angiogenesis and abnormal cell differentiation observed in the synovial membrane. The study also revealed the genetic effect of TXNDC5 on RA and AS risk.

## Abbreviations

AS: ankylosing spondylitis; DMARD: disease-modifying anti-rheumatic drug; KLH: keyhole limpet hemocyanin; LD: linkage disequilibrium; MAF: minor allele frequencies; NSAID: non-steroidal anti-inflammatory drugs; NSV: nonsegmental vitiligo; OA: osteoarthritis; PDI: disulfide isomerase domain; RA: rheumatoid arthritis; SDS-PAGE: sodium dodecyl sulphate polyacrylamide gel electrophoresis; SLE: systemic lupus erythematosus; SNP: single nucleotide polymorphism; TXNDC5: Thioredoxin domain containing 5; UTR: untranslational regions

## Competing interests

The authors declare that they have no competing interests.

## Authors' contributions

XC designed and executed the study and prepared the manuscript. JP and KF performed the genotyping. YZ and LW performed the Western blots and real time PCR. XY collected tissue samples. All authors have read and approved the final manuscript for publication.

## Supplementary Material

Additional file 1 **Supplementary materials and methods**. This table summarizes the clinical data of patients with RA, OA and AS. Click here for file

Additional file 2 **Supplementary results**. This table provides the raw microarray data to perform association, LD and haplotype analysis. We genotyped 96 SNPs across the TXNDC5 gene from 267 Han Chinese patients with RA, 51 patients and 160 control individuals. All SNPs yielded genotype data, and the study sample success rate was 99.1%. Click here for file
