# Supplementary material for: Investigating a pathogenic role for TXNDC5 in rheumatoid arthritis
Source: Arthritis Res Ther. 2011 Jul 29;13(4):R124. doi: 10.1186/ar3429 (PMC3239364; doi:10.1186/ar3429)
Supplement: Additional file 1 — Supplementary materials and methods. This table summarizes the clinical data of patients with RA, OA and AS. [file ar3429-S1.DOC]

**Additional file 1 Clinical data on patients with RA,OA and** AS

| Diagnosis | Patient  No. | Sex | Age (years) | HLA-  B27 | RF | anti-  CCP  (U/ml) | ESR  (mm/h) | CRP  (mg/Dl) | Disease duration  (years) | NSAIDs treatment | DMARDs  treatment |
| --- | --- | --- | --- | --- | --- | --- | --- | --- | --- | --- | --- |
| AS | 1 | male | 35 | + | - | - | 46 | 34 | 3 | yes | yes |
| AS | 2 | male | 29 | + | - | - | 7 | 6 | 12 | yes | yes |
| AS | 3 | male | 24 | + | - | - | 16 | 6 | 5 | yes | yes |
| AS | 4 | male | 31 | + | - | - | 45 | 6 | 6 | yes | yes |
| AS | 5 | male | 36 | + | - | - | 40 | 6 | 6 | yes | yes |
| AS | 6 | male | 37 | + | - | - | 24 | 5 | 8 | yes | yes |
| AS | 7 | male | 13 | + | - | - | 62 | 8 | 7 | yes | yes |
| AS | 8 | female | 48 | + | - | - | 58 | 10 | 5 | yes | yes |
| AS | 9 | female | 28 | + | - | - | 10 | 6 | 10 | yes | yes |
| AS | 10 | female | 54 | + | - | - | 12 | 6 | 10 | yes | yes |
| RA | 11 | female | 30 | ND | 100 | 209 | 80 | 6 | 4 | yes | yes |
| RA | 12 | female | 58 | ND | 160 | 279 | 72 | 192 | 9 | yes | yes |
| RA | 13 | female | 65 | ND | 496 | 218 | 66 | 153 | 7 | yes | yes |
| RA | 14 | female | 41 | ND | 78 | 168 | 39 | 10 | 3 | yes | yes |
| RA | 15 | female | 64 | ND | 200 | 169 | 61 | 10 | 5 | yes | yes |
| RA | 16 | male | 53 | ND | 107 | 171 | 55 | 20 | 3 | yes | yes |
| RA | 17 | female | 49 | ND | 160 | 227 | 50 | 96 | 4 | yes | yes |
| RA | 18 | male | 50 | ND | 1280 | 394 | 84 | 153 | 6 | yes | yes |
| RA | 19 | female | 53 | ND | 599 | 171 | 31 | 6 | 7 | yes | yes |
| RA | 20 | female | 48 | ND | 107 | 92 | 13 | 60 | 10 | yes | yes |
| OA | 21 | male | 40 | ND | - | ND | 10 | 6 | 6 | yes | - |
| OA | 22 | female | 62 | ND | - | ND | 68 | 48 | 10 | yes | - |
| OA | 23 | female | 53 | ND | - | ND | 20 | 6 | 8 | yes | - |
| OA | 24 | female | 60 | ND | - | ND | 22 | 6 | 8 | yes | - |
| OA | 25 | male | 66 | ND | - | ND | 24 | 10 | 6 | yes | - |
| OA | 26 | female | 72 | ND | - | ND | 24 | 6 | 5 | yes | - |
| OA | 27 | male | 65 | ND | - | ND | 5 | 10 | 11 | yes | - |
| OA | 28 | male | 72 | ND | - | ND | 8 | 6 | 13 | yes | - |
| OA | 29 | male | 68 | ND | - | ND | 24 | 6 | 7 | yes | - |
| OA | 30 | male | 67 | ND | - | ND | 10 | 20 | 6 | yes | - |

ND: no data available
